# Supplementation of Rumen-Protected Methionine to Heat-Stressed Transition Cows Impacts Mammary Gland Cell Dynamics and Gene Expression

**DOI:** 10.3390/ani16172770

**Published:** 2026-09-03

**Authors:** Brittney D. Davidson, Maverick C. Guenther, Julie E. Duclos, Marta Sfulcini, Danielle N. Sherlock, Marion Boutinaud, Jimena Laporta

**Affiliations:** 1 Department of Animal and Dairy Sciences, University of Wisconsin-Madison, Madison, WI 53706, USA; brittney.davidson@usda.gov (B.D.D.); mcguenther@wisc.edu (M.C.G.); 2 INRAE, Institut Agro Rennes Angers, PEGASE, 35590 Saint-Gilles, France; julie.duclos@inrae.fr (J.E.D.); marion.boutinaud@inrae.fr (M.B.); 3 Department of Animal Science, Food and Nutrition, Universitá Cattolica del Sacro Cuore, 29100 Piacenza, Italy; marta.sfulcini@unicatt.it; 4 Adisseo USA Inc., Alpharetta, GA 30022, USA; danielle.sherlock@adisseo.com

**Keywords:** transition, epithelial integrity, mammary remodeling, hyperthermia

## Abstract

Heat stress (HS) during the transition period disrupts metabolic adaptation and mammary gland (MG) remodeling. Methionine, a rate-limiting amino acid, supports cellular growth and stress responses through one-carbon metabolism pathways. We evaluated whether rumen-protected methionine (RPM) supplementation during the transition period preserves MG structure and function in heat-stressed multiparous Holstein cows. We hypothesized that RPM would mitigate the negative effects of HS on MG microstructure, gene expression, and cellular turnover. A total of 53 multiparous Holstein cows were enrolled at dry-off and assigned to diets with or without RPM (MET, CON). Four weeks prepartum, all MET and half of CON cows were fitted with electric heat blankets to induce HS (METHS, CONHS), while remaining CON cows were thermoneutral (CONTN). MG biopsies (8 and 28 days in milk; DIM) and milk samples were collected to assess microstructure, cellular turnover, gene expression, and mammary epithelial integrity. HS appeared to impair MG remodeling, with fewer but larger alveoli and a thicker epithelial layer, with a slight increase in epithelial cell proliferation, increased occludin fluorescence intensity, and increased milk concentration of K^+^. Meanwhile, RPM under HS increased stromal proliferation, lower occludin fluorescence intensity, and a reduced Na^+^:K^+^ ratio, indicating overall improved mammary epithelial integrity. Additionally, RPM partially promoted greater alveolar organization early postpartum and reduced lumen expansion, with coordinated changes in one-carbon metabolism, amino acid transport, and mTOR-related pathways. Overall, HS disrupts MG cellular dynamics, whereas close-up methionine supplementation partially improves mammary adaptability under chronic transition period HS.

## Simple Summary

Dairy cows undergo physiological changes around calving as they shift from non-lactating and pregnant to lactating. Heat stress during this time intensifies these challenges by reducing intakes, weakening the immune system, lowering fertility, and decreasing milk production. As global temperatures rise, managing heat stress is challenging for producers. This study explored whether methionine supplementation, an amino acid involved in cell growth and stress response, could help protect the mammary gland during heat stress. A total of 53 cows were either fed a control diet or one supplemented with rumen-protected methionine. To induce heat stress, half the control cows and all methionine-fed cows were fitted with an electric heat blanket; the remaining cows were kept at thermoneutrality. After calving, milk samples and tissue biopsies were collected to examine mammary gland function. Heat-stressed cows showed disrupted mammary tissue, with fewer but enlarged alveoli, increased leakage of metabolites from blood into the milk, suggesting compromised epithelial remodeling and weakened tight junctions, ultimately reducing the capacity for milk synthesis. Methionine supplementation partially attenuated these heat-induced effects and was associated with altered gene expression related to growth, nutrient utilization, and one-carbon pathways. Overall, heat stress harms mammary development, but methionine may help cows cope during this critical period.

## 1. Introduction

Heat stress (HS), characterized by increases in both ambient temperatures and relative humidity, is a major environmental and economic challenge affecting the dairy industry and occurs when heat accumulation surpasses the animals’ ability to maintain thermal balance through heat dissipation [1]. These HS conditions lead to decreased DMI [2], elevated thermal indices [3,4,5], a suppression of the immune system and fertility [6,7], in addition to a reduction in milk and milk component yields [4,5,8,9]. To partially overcome negative consequences of HS, producers typically adopt heat abatement systems including shade provision, as well as fans and sprinklers to promote evaporative cooling. However, as temperature prediction models have estimated that global temperatures are on the rise [10,11], increasing drought and water scarcity are expected to limit the availability of water, threatening the efficacy and sustainable use of these water-based cooling strategies.

Heat stress impacts dairy cows across all stages of development and production. Of major concern are the effects during the transition period, which is marked by extensive metabolic adaptations, increased disease incidence, and mammary gland remodeling as the tissue goes from a dry, non-lactating state to a lactating state following parturition [12,13,14,15]. Additionally, during this peri-parturition period, dairy cows experience some degree of negative energy balance due to a reduction in DMI and a concomitant increase in nutrient requirements to support both fetal growth and the onset of lactation [15]. Although reduced DMI accounts for a portion of the production losses observed under HS (estimated at ~35–50%), it does not fully explain these responses, suggesting direct effects of HS on key tissues and organs, including the mammary gland [16,17]. Collectively, these challenges further restrict and redirect the supply of amino acids during a time when fetal, metabolic, and mammary demands are increasing. An insufficient amino acid supply reduces fetal growth, decreases milk protein synthesis, and increases lipid and amino acid mobilization to be taken up by the liver [8,14,18].

Over the past several decades, amino acid supplementation has received considerable attention and has been shown to play important roles in modulating the expression of genes encoding milk proteins, enhancing milk protein synthesis, supporting immune regulation, maintaining epithelial integrity, and facilitating metabolic adaptation. Specifically, methionine, often considered a rate-limiting amino acid for milk protein synthesis, has been the primary focus of amino acid supplementation studies [19,20]. Consistently, supplementation of rumen-protected methionine (RPM) in transition cows has been associated with increased DMI, milk yield, and milk protein concentration and yield [21,22,23,24,25,26]. Beyond serving as a substrate for protein synthesis, methionine also regulates key signaling pathways involved in cellular growth and protein translation through one-carbon metabolism. In particular, methionine is metabolized to S-adenosylmethionine, a cellular metabolite that reflects nutrient availability and can stimulate the mechanistic target of rapamycin (mTOR) pathway [27,28]. Activation of mTOR is thought to influence protein synthesis, cell proliferation, and tissue remodeling in mammary epithelial cells [29]. Additionally, SAM supports one-carbon metabolism by contributing to methylation reactions, antioxidant production, and gene regulation; processes that influence cellular turnover and stress resilience [30].

Understanding how amino acid availability regulates both the structural and functional aspects of the mammary gland tissue is essential for elucidating the mechanisms by which nutritional interventions, such as methionine supplementation, may alleviate HS-induced alterations. Herein, we evaluated the effects of feeding RPM to multiparous Holstein cows during the transition period under induced HS on mammary gland microstructure and cellular activity. We hypothesized that increasing dietary RPM during late gestation and early lactation would mitigate the negative effects of HS on MG microstructure, gene expression, and cellular activity, relative to thermoneutral counterparts.

## 2. Materials and Methods

### 2.1. Animals and Experimental Design

All animal procedures were approved by the Institutional Animal Care and Use Committee at the University of Wisconsin-Madison and conducted in the summer and fall of 2022 under protocol #A006505. The experimental design, diets fed and composition, and transition cow management are described in detail in a previously published study focusing on health and production outcomes [26]. Briefly, 53 multiparous Holstein dams were dried-off approximately 50 d prior to their expected calving date. Following dry-off, cows were moved to tie-stalls and assigned to either a control diet (CON, 2.2% Met of MP, *n* = 36) or the same diet supplemented with additional Smartamine^®^M (MET, 2.6% Met of MP, *n* = 17, Adisseo Inc., Antony, France). Beginning at 4 weeks (28 d) before the expected calving date, all MET-supplemented cows and half of the CON cows were fitted with an electric heat blanket (EHB, Thermotex Therapy Systems Ltd., Calgary, AB, Canada) to induce heat stress responses. The remaining CON cows were maintained at thermoneutrality (TN). This resulted in three treatments: CON diet without EHB (CONTN, *n* = 19), CON diet with EHB (CONHS, *n* = 17), and MET-supplemented cows with EHB (METHS, *n* = 17). Dietary (−42 to +28 d relative to calving, total duration of 70 d) and thermal (−28 to +28 d relative to calving, total duration of 56 d) treatments were applied throughout the transition period. All cows were housed in a tie-stall barn equipped with evaporative cooling (tunnel ventilation and cooling cells), with no direct cooling applied at the cow level. During the experimental period, mean ambient temperature, relative humidity, and temperature-humidity index averaged 17.1 ± 4.5 °C, 66.9 ± 17.7%, and 62 ± 7.0, respectively [26]. Cows had ad libitum access to water and feed, which was offered once daily at a 5–10% refusal rate. The two experimental diets (CON and MET; Smartamine^®^M, Adisseo Inc., France) were formulated to be identical in composition and nutrient contents, differing only in the inclusion of RPM [26]. The EHB induced mild HS, as evidenced by increased respiration rate and skin temperature throughout the transition period, and elevated rectal temperature particularly after calving. Moreover, RPM supplementation increased circulating methionine concentrations during the transition period (27 vs. 39 μM) [26].

### 2.2. Mammary Parenchyma Tissue Collection and Histological Analyses

Mammary gland biopsies of parenchyma tissue were harvested at 8 (*n* = 6–8/treatment) and 28 (*n* = 7–9/treatment) days following parturition. A detailed description of the mammary tissue biopsy protocol and tissue processing can be found in [31]. Immediately after collection, mammary tissue was washed in sterile PBS and placed in plastic cassettes in 10% neutral-buffered formalin for 20 h at room temperature. After overnight incubation, cassettes were transferred to sterile PBS and stored at 4 °C until paraffin embedding. Tissues were serial sectioned at a thickness of 5 µm onto glass slides. Hematoxylin and eosin staining was performed to visualize mammary microstructure according to standard protocols. Four representative photomicrographs were captured (20× objective lens) per slide with the BZ-X800 Keyence microscope (Keyence Corporation, Osaka, Japan) to quantify the number of alveoli, average number of mammary epithelial cells (MEC) per alveoli, the alveoli outer area, the alveoli inner luminal area, inner-to-outer alveolar area ratio, and the mammary epithelial cell layer area in the ImageJ software (version 1.54m) [32].

For occludin and cell turnover assays, sections were deparaffinized with xylene (2 washes; 5 min each), and rehydrated in a graded ethanol series (100%, 100%, 75%, 50%; 5 min each) and a final wash in pure H_2_O (1 min). Occludin protein abundance was assessed by immunofluorescence intensity (*n* = 6/treatment group). Sections underwent antigen retrieval in citrate buffer at 95 °C for 20 min in a water bath, followed by blocking using 5% normal goat serum to reduce nonspecific binding. Samples were incubated with a recombinant anti-Occludin primary antibody (Abcam, ab216327, Cambridge, UK) at 1:200 in 1% bovine serum albumin, followed by incubation with Goat anti-Mouse secondary antibody (Alexa Fluor 555, A21429, Invitrogen by Thermo Fisher Scientific, Loughborough, Leicestershire, UK) at 1:250 in and cover slipped using EverBrite™ Mounting Medium containing DAPI to visualize nuclei (Biotium, Fremont, CA, USA). Negative controls, prepared by omitting the primary antibody, were included for each staining to confirm labeling specificity. Six microphotographs per ID per time point (20× objective lens, BZ-X800 Keyence, Keyence Corporation, Osaka, Japan) were captured for occludin. Fluorescence intensity in the tissue was quantified using two excitation wavelengths to measure both occludin fluorescence (BZ-X Analyzer Software, version 1.1.30) and the number of DAPI-stained nuclei (ImageJ-Fiji software, version 2.14.0). The total fluorescence intensity was then divided by the number of nuclei, determined from DAPI-positive nuclei, to obtain the mean fluorescence per nucleus, which was further divided by 100,000 for graphing visualization.

Mammary cell death (*n* = 6/group) was quantified via immunohistochemistry using the Apoptag Plus in situ Apoptosis Kit (EMD Millipore Corp, Burlington, VT, USA; S7101) with modifications to the manufacturer’s protocol as described by [33]. Six representative photomicrographs per animal per time point (20× objective lens, BZ-X800 Keyence, Keyence Corporation, Osaka, Japan) were captured. The total number of cells was obtained using the BZ-X Analyzer Software (version 1.1.30), and the number of positive cells was manually counted using ImageJ. Mammary cell proliferation (*n* = 6/treatment group) was quantified via immunohistochemistry staining of the Ki67 nuclear proteins previously described by [31]. Briefly, mouse anti-human Ki67 (1:100, DAKO #M7240, clone MIB-1, Glostrup, Denmark) was incubated on mammary sections for 60 min, and staining was visualized using Mach 2 Mouse HRP polymer (Biocare Medical, Walnut Creek, CA, USA; #MHRP520L). Six representative microphotographs (20× objective lens, Keyence microscope, Keyence Corporation, Osaka, Japan) were captured. For each immunohistochemical assay, each nucleus was labeled as positive (brown) or negative (blue) in each location (i.e., alveolar or stomal). The proportion of Ki67 or TUNEL-positive nuclei was calculated as the total number of positive nuclei across all fields within each location divided by the total number (positive and negative) of nuclei.

### 2.3. Mammary Gland RNA Extraction and Gene Expression

A portion of the mammary tissue biopsies was snap-frozen in liquid nitrogen immediately after collection and stored at −80 °C for downstream RNA isolation. Total RNA was extracted from mammary tissue at 8 (*n* = 6–7/group) and 28 (*n* = 6–7/group) DIM using the Purelink RNA Mini Kit (Cat# 121183025; Invitrogen, Carlsbad, CA, USA) according to the manufacturer’s instructions using TRIzol Reagent (Cat# 15596026; Invitrogen, Carlsbad, CA, USA) for tissue lysis. The concentration and quality of RNA were measured using the Implen Nanophotometer (Munich, Germany), and 1 μg of RNA was utilized for reverse transcription with iScript Reverse Transcription Supermix (Bio-Rad, Cat# 1708841, Hercules, CA, USA). Real-time-qPCR was performed using a CFX96 Real-Time PCR detection System (Bio-Rad, Hercules, CA, USA) with reaction mixtures and cycling conditions performed as previously described [34]. Positive (housekeeping primer mix with a pool of concentrated cDNA) and negative (housekeeping primer mix with water) controls were included in each plate, and samples were run in duplicate. Genes of interest included those associated with tight junctions (*ZO-1*, *OCLN*), milk protein synthesis (*LALBA*, *CSN1S1*, *CSN1S2*, *CSN2*, and *CSN3*), amino acid transport (*SLC7A5* and *SLC3A2*), one-carbon metabolism, methylation, and epigenetic regulation (*MTR*, *MAT*, *AHCY*, *GNMT*, *DNMTI*, *DNMT3A*), and mTOR signaling (*MTORC1*, *AKT1*, *ULK1*, *RPS6*, *RPS6KB1*, and *EIF4EBP1*). Primer sequences are in Appendix A Table A1. Using the ΔΔCT method, the geometric mean of three housekeeping genes (*UXT*, *ACTB*, and *KRT8*) was used to normalize the relative gene expression. Values were calculated so that positive ΔΔCT indicated greater gene expression relative to the housekeeping genes and negative ΔΔCT indicated downregulation. This sign convention was intentionally applied for interpretive clarity. Expression of these reference genes was stable across timepoints and treatments. The stability of the housekeeping genes was evaluated separately for each timepoint using an online tool [35] based on the geNorm algorithm [36]. Individually, all three selected housekeeping genes in each tissue type had an M value ≤ 1.5, suggesting adequate stability across treatments. The combination of selected housekeeping genes at DIM 8 and DIM 28 had an average M value of 1.48 and 1.47, respectively.

### 2.4. Milk Collections and Mineral Composition Assessment

Four-quarter composite milk samples were collected at the milking parlor on days 4, 7, 10, 13, 16, 19, 22, 25, and 28 of lactation (*n* = 8–9/group). To analyze the milk mineral composition (sodium and potassium) closest to the time points of the mammary parenchyma tissue harvest, composite milk samples were collected on d 7 and 28 (morning prior to mammary tissue biopsies) in 15 mL tubes. Samples were stored at −20 °C until further analysis. The Na^+^/K^+^ ratio in milk is known to be inversely correlated with mammary epithelial integrity during lactation [37]. This relationship was estimated by measuring the milk minerals using inductively coupled plasma atomic emission spectrometry (ICP-AES; 5110 Agilent Technology, Les Ulis, France) previously reported by [38]. Samples were first diluted in H_2_0 and then 2.5 mL of 0.01% Triton X100 (Sigma-Aldrich, Saint-Quentin Fallavier, France) and 7.5 mL of 65% nitric acid were added. Analyses were performed according to manufacturer instructions using calibration standards for ICP-AES Certipur Potassium and Sodium 1000 µg/mL (Agilent Technology, Les Ulis, France) and a standard milk sample ERM-BD151 (European Reference Materials, milk sample with guaranteed contents, European Commission Directorate-General JRC—Joint Research Centre Brussels, Belgium).

### 2.5. Statistical Analysis

All statistical analyses were performed in SAS (version 9.4, SAS Institute Inc., Cary, NC, USA). Residuals were tested for homogeneity of variance using Levene’s test and for normality using the Shapiro–Wilk statistic (PROC UNIVARIATE). Levene’s test was used to confirm equal residual variances across treatments and the Shapiro–Wilk statistic verified that residuals were normally distributed, ensuring the assumptions of the statistical models were met. Mammary gland microstructure, gene expression, immunofluorescence, and milk minerals data were analyzed using a generalized linear mixed model, with treatment (CONTN, CONHS, METHS) as a fixed effect and animal ID nested within treatment as a random effect. Treatment effects on both DIM 8 and DIM 28 were analyzed separately because different cows were sampled at each time point. The number of images/animal ID was included as a repeated statement, and specifically for occludin staining, date was included as a covariate to account for potential laboratory variation. Cellular turnover data (Ki67 and TUNEL) were analyzed using PROC GLIMMIX with a binomial distribution and logit link function, modeling the proportion of positive cells relative to total cells within stromal, epithelial, and total mammary tissue compartments. Animal ID within image replicate was included as a random effect. Results are presented as least squares means ± standard error. Pre-planned pairwise comparisons of interest included CONTN vs. CONHS and CONHS vs. METHS. To note, a formal comparison between CONTN and METHS is not statistically valid because these groups differ in both diet and environmental condition. Without an MET-supplemented cow without an EHB (MET-TN) group in the present study, the effects of methionine and heat stress are confounded, preventing an interpretable contrast. Significance was declared at *p* ≤ 0.05 and tendencies at 0.05 < *p* ≤ 0.10.

## 3. Results

### 3.1. Mammary Gland Microstructure

**Figure 1:**
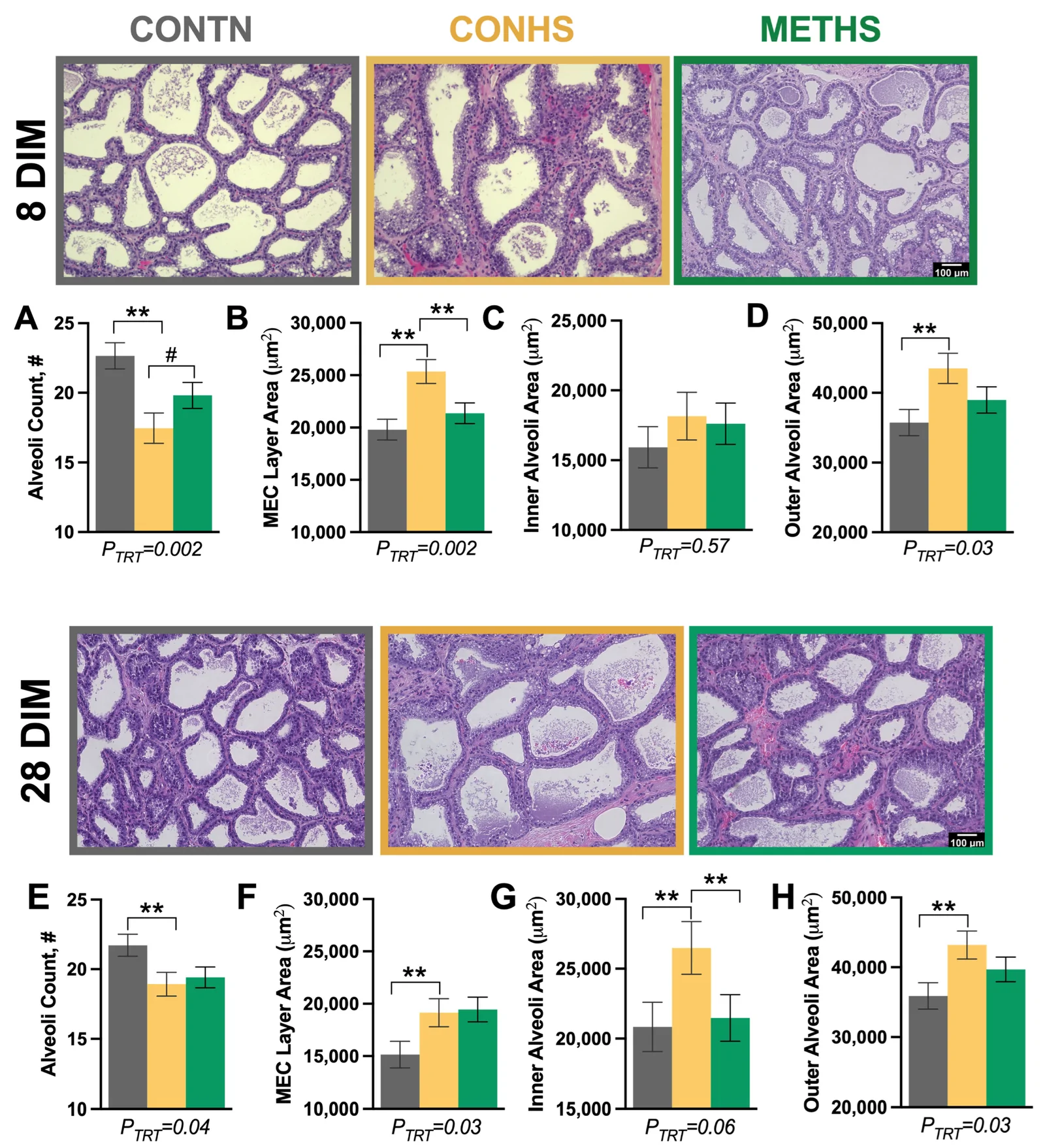
Mammary gland biopsies (*n* = 6–9/group) were collected at 8 and 28 DIM to evaluate alveolar morphology, including (**A**,**E**) the number of alveoli, (**B**,**F**) the area of the mammary epithelial cell layer within the alveoli, (**C**,**G**) the inner or luminal area of the alveoli, and (**D**,**H**) the outer area of the alveoli. Multiparous cows were assigned to one of three treatments: thermoneutral conditions with a control diet (CONTN; grey bars; *n* = 8 at 8 DIM; *n* = 8 at 28 DIM), artificially induced heat stress with a control diet (CONHS; yellow bars; *n* = 6 at 8 DIM; *n* = 7 at 28 DIM), or artificially induced heat stress with rumen-protected methionine supplementation (METHS; green bars; Smartamine^®^ M Adisseo Inc., France; *n* = 8 at 8 DIM; *n* = 9 at 28 DIM) providing 2.6% Met of metabolizable protein compared with 2.2% in control diets. Diets (CON vs. MET) were fed from −6 weeks prepartum through 4 weeks postpartum, and environmental treatments (TN vs. HS) were applied from −4 weeks prepartum through 4 weeks postpartum. Heat stress was induced using electric heat blankets (Thermotex Therapy Systems Ltd., Calgary, AB, Canada). Representative histological images (20× objective lens; scale bar = 100 µm) are shown above each treatment. Data are presented as least squares means ± SEM and treatment effects (*P_TRT_*) are reported within each graph. Only significant (**; *p* ≤ 0.05) and trending (#; 0.05 < *p* ≤ 0.10) pre-planned pairwise comparisons (CONTN vs. CONHS; CONHS vs. METHS) are indicated. The average number of mammary epithelial cells per alveolus and the inner-to-outer alveolar area ratio were analyzed but did not differ among treatments and are therefore not shown.

At 8 DIM (*n* = 6–8/treatment), there were no differences between treatments in the average number of mammary epithelial cells per alveoli (*P_TRT_* = 0.62; 47.2 vs. 50.7 vs. 50.4 ± 2.9 MEC; CONTN vs. CONHS vs. METHS, respectively), the inner (luminal) size of the alveoli (*P_TRT_* = 0.57; 15,929 vs. 18,155 vs. 17,613 ± 1549 µm^2^; CONTN vs. CONHS vs. METHS, respectively; Figure 1C), or the inner to outer alveoli size ratio (*P_TRT_* = 0.30; 0.39 vs. 0.35 vs. 0.39 ± 0.02; CONTN vs. CONHS vs. METHS, respectively). There was a significant treatment effect for alveoli number, mammary epithelial cell layer area, and outer alveoli size (*P_TRT_* ≤ 0.03). Compared to CONTN, CONHS mammary tissue had fewer alveoli (*p* = 0.0005; 23 vs. 17 ± 1 alveoli; CONTN vs. CONHS, respectively; Figure 1A), larger MEC layers (*p* = 0.0004; 19,802 vs. 25,355 ± 1066 µm^2^; Figure 1B), and larger alveoli (measured at the outer boundary; *p* = 0.008; 35,731 vs. 43,510 ± 2014 µm^2^; Figure 1D). Additionally, compared to METHS, CONHS mammary tissue tended to have fewer alveoli (*p* = 0.10; 20 vs. 17 ± 1 alveoli; METHS vs. CONHS, respectively; Figure 1A) and had larger mammary epithelial cell layers (*p* = 0.01; 21,366 vs. 25,355 ± 1066 µm^2^; Figure 1B). The outer area of the alveoli was not different between METHS and CONHS cows (*p* = 0.12; 38,979 vs. 43,510 ± 2014 µm^2^; Figure 1D).

At 28 DIM (*n* = 7–9/treatment), no differences between treatments were captured in the average number of MEC per alveoli (*P_TRT_* = 0.19; 44.7 vs. 48.0 vs. 44.6 ± 1.4 MEC; CONTN vs. CONHS vs. METHS, respectively) or for the inner to outer alveoli size ratio (*P_TRT_* = 0.34; 0.57 vs. 0.54 vs. 0.50 ± 0.2; CONTN vs. CONHS vs. METHS, respectively). There was a significant treatment effect for alveoli number, mammary epithelial cell layer, and outer alveoli area (*P_TRT_* ≤ 0.04) and a tendency for a treatment effect on inner alveoli area (*P_TRT_* = 0.06). Compared to CONTN, CONHS mammary tissue had fewer alveoli (*p* = 0.02; 22 vs. 19 ± 1 alveoli; CONTN vs. CONHS, respectively; Figure 1E), larger MEC layers (*p* = 0.03; 15,143 vs. 19,145 ± 1303 µm^2^; Figure 1F), larger alveolar lumens (*p* = 0.03; 20,839 vs. 26,492 ± 1826 µm^2^; Figure 1G), and larger outer alveoli measurements (*p* = 0.01; 35,909 vs. 43,188 ± 1953 µm^2^; Figure 1H). Moreover, compared to METHS, CONHS alveoli lumens were larger (*p* = 0.05; 21,479 vs. 26,492 ± 1776 µm^2^; METHS vs. CONHS, respectively; Figure 1G) on day 28 of lactation. The number of alveoli (*p* = 0.67; 19 vs. 19 ± 1 alveoli; Figure 1E), mammary epithelial cell layer area (*p* = 0.86; 19,452 vs. 19,145 ± 1258 µm^2^; Figure 1F), and the outer alveoli area (*p* = 0.20; 39,698 vs. 43,188 ± 1897 µm^2^; Figure 1H) was not different between METHS and CONHS cows.

### 3.2. Mammary Gland Gene Expression

**Figure 2:**
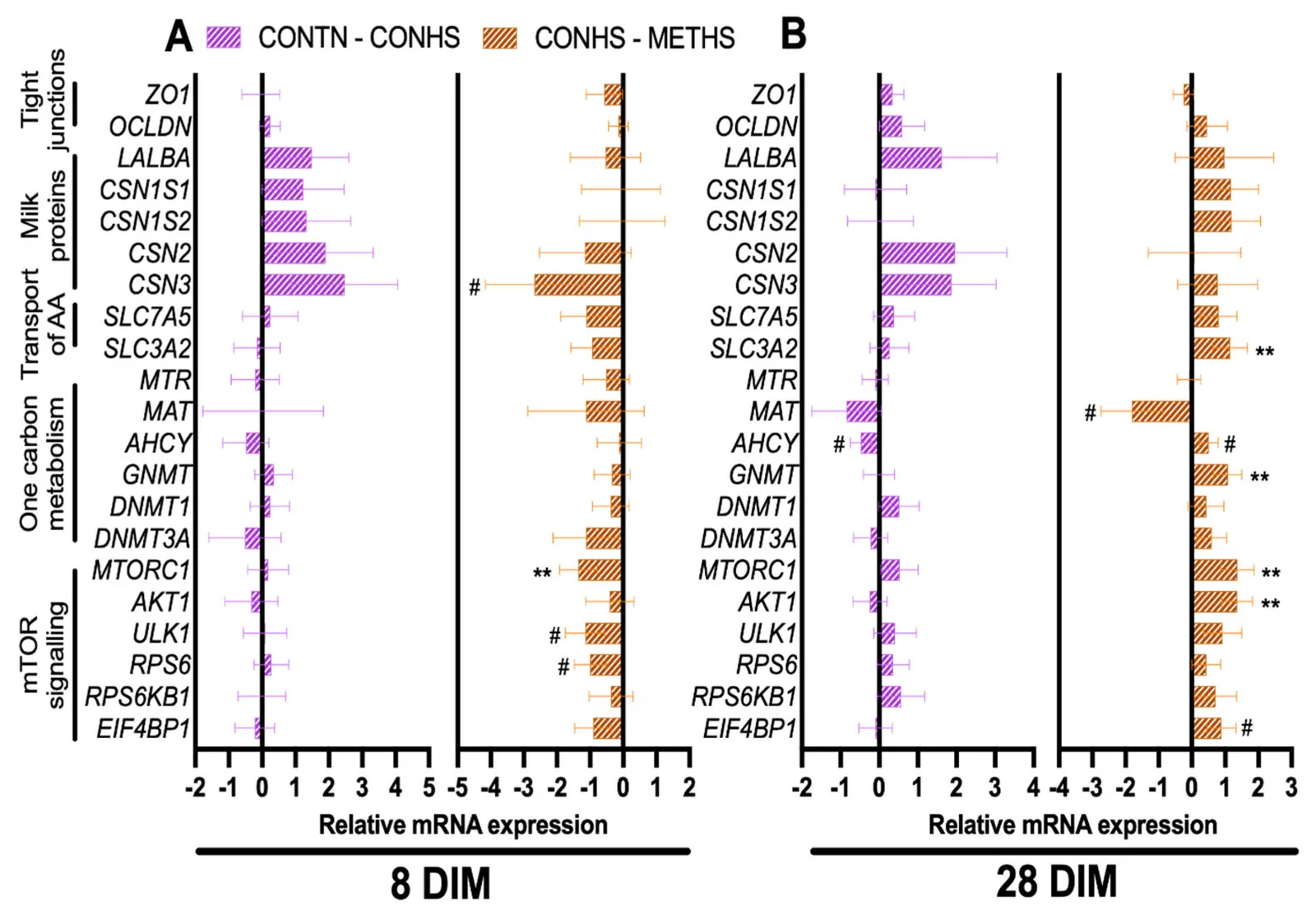
Relative mRNA expression in mammary tissue at 8 and 28 DIM (*n* = 6–7/group) from multiparous cows assigned to thermoneutral conditions with a control diet (CONTN; *n* = 6 at 8 DIM; *n* = 7 at 28 DIM), artificially induced heat stress with a control diet (CONHS; *n* = 6 at 8 DIM; *n* = 6 at 28 DIM), or artificially induced heat stress with rumen-protected methionine supplementation (METHS; Smartamine^®^ M, Adisseo Inc., France; *n* = 7 at 8 DIM; *n* = 6 at 28 DIM), providing 2.6% Met of metabolizable protein compared with 2.2% in control diets. Expression was evaluated for 21 genes involved in tight junctions (*ZO-1*, *OCLN*), milk protein synthesis (*LALBA*, *CSN1S1*, *CSN1S2*, *CSN2*, and *CSN3*), amino acid transport (*SLC7A5* and *SLC3A2*), one-carbon metabolism and methylation pathways and regulation (*MTR*, *MAT*, *AHCY*, *GNMT*, *DNMTI*, *DNMT3A*), and mTOR signaling pathway (*MTORC1*, *AKT1*, *ULK1*, *RPS6*, *RPS6KB1*, and *EIF4EBP1*). Data is presented as relative mRNA expression (ΔΔCt), where positive and negative values indicate upregulation and downregulation of ((**A**); purple bars) CONHS relative to CONTN or ((**B**); brown bars) METHS relative to CONHS. Significance (**) was declared at *p* ≤ 0.05, and # indicates a tendency at 0.05 < *p* ≤ 0.10.

At 8 DIM (*n* = 6–7/group), the relative mRNA expression for all genes tested was not different for the CONHS cows, relative to CONTN (*p* ≥ 0.14; Figure 2A). Among the mTOR signaling pathway genes, mechanistic target of rapamycin complex 1 (*MTORC1*) was significantly downregulated in METHS (*p* = 0.03; Figure 2A), while unc-51 like kinase 1 (*ULK1*; *p* = 0.07; Figure 2A) and ribosomal protein S6 (*RPS6*; *p* = 0.06; Figure 2A) showed a trend toward downregulation in METHS, relative to CONHS cows. The milk protein gene, casein kappa (*CSN3*), tended to be downregulated in METHS cows, relative to CONHS (*p* = 0.09; Figure 2A).

Furthermore, an intermediate pathway of the methylation cycle, adenosylhomocysteine hydrolase (*AHCY*), tended to be downregulated in the CONHS cows at 28 DIM (*n* = 6–7/group; *p* = 0.10; Figure 2B), relative to CONTN. Relative to CONHS, the relative expression of solute carrier family 3 member 2 (*SLC3A2*) was upregulated in METHS cows at 28 DIM (*p* = 0.04; Figure 2B). Within the one-carbon metabolism genes, methionine adenosyltransferase (*MAT*) tended to be downregulated in METHS (*p* = 0.07; Figure 2B), adenosylhomocysteine hydrolase (*AHCY*) tended to be upregulated in METHS (*p* = 0.09; Figure 2B), and glycine N-methyltransferase (*GNMT*) was upregulated in METHS (*p* = 0.02; Figure 2B), relative to CONHS cows. Moreover, at 28 DIM, METHS had an upregulation of mechanistic target of rapamycin complex 1 (*MTORC1*; *p* = 0.01; Figure 2B) and protein kinase B (*AKT1*; *p* = 0.01; Figure 2B) and showed a trend toward upregulated eukaryotic translation initiation factor 4E-binding protein 1 (*EIF4EBP1*; *p* = 0.06; Figure 2B), relative to CONHS cows.

### 3.3. Mammary Gland Barrier Function and Permeability

**Figure 3:**
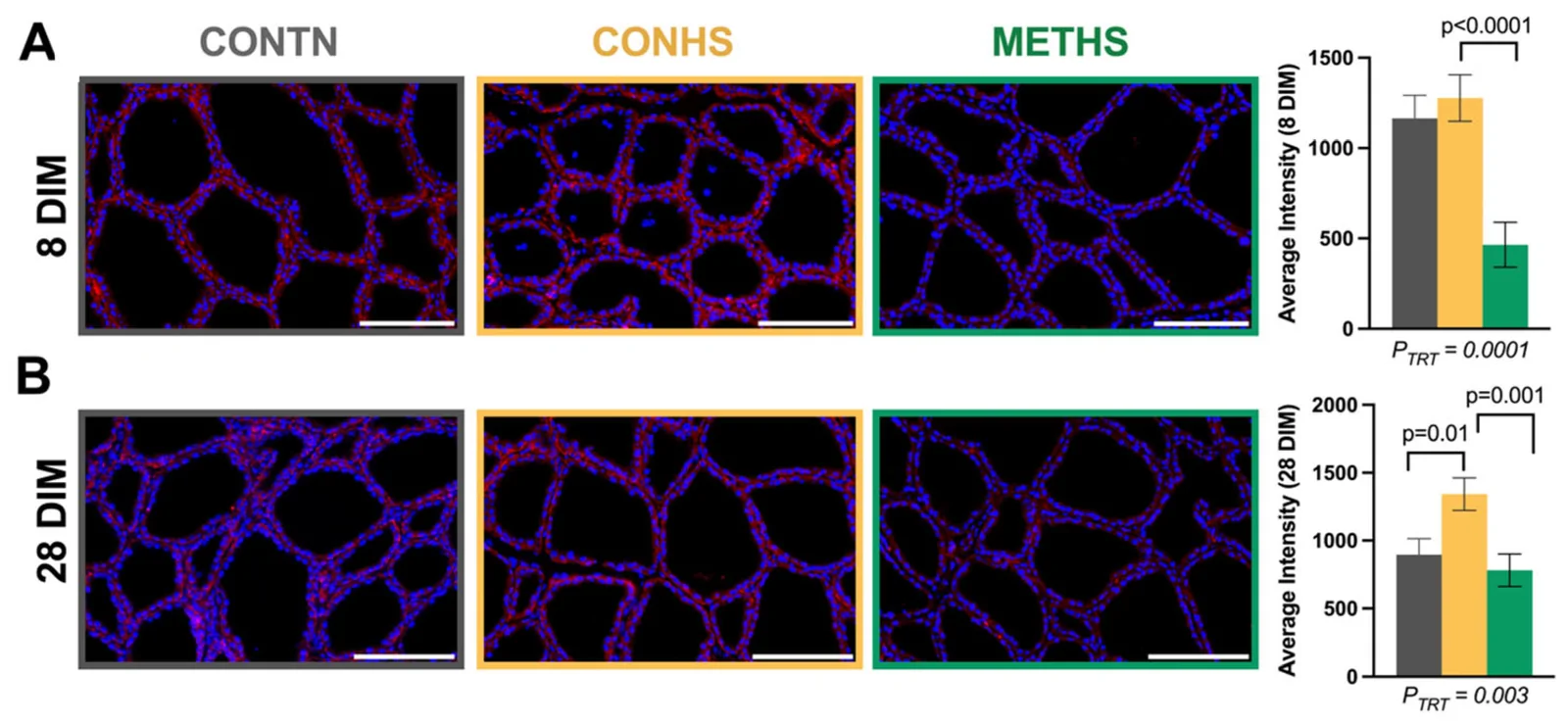
Representative immunofluorescence of the tight junction protein occludin in mammary tissue at 8 (**A**) and 28 DIM (**B**) from multiparous cows assigned to thermoneutral conditions with a control diet (CONTN; grey bars; *n* = 6), heat stress with a control diet (CONHS; yellow bars; *n* = 6), or heat stress with rumen-protected methionine supplementation (METHS; green bars; *n* = 6; Smartamine^®^ M, Adisseo Inc., France), providing 2.6% Met of metabolizable protein compared with 2.2% in control diets. Images were captured at a 20× objective lens. Fluorescence intensity was quantified using two excitation wavelengths and normalized to the number of nuclei (DAPI-positive) to obtain mean fluorescence per nucleus. Values were scaled (÷100,000) for visualization. Data are presented as least squares means ± SEM. Treatment effects (*P_TRT_*) are reported within each panel, and pre-planned pairwise comparisons of interest (CONTN vs. CONHS and CONHS vs. METHS) are shown with exact *p*-values.

There was a significant treatment effect on 8 DIM (*n* = 6/group; *P_TRT_* < 0.0001). Specifically, the average occludin intensity did not differ between CONTN and CONHS (*p* = 0.54; 1164.6 vs. 1277.0 ± 127.8; CONTN vs. CONHS, respectively; Figure 3A). Compared to CONHS, METHS cows had significantly lower occludin fluorescence intensity (*p* < 0.0001; 464.7 vs. 1277.0 ± 126.5; METHS vs. CONHS, respectively; Figure 3A). Moreover, there was a significant treatment effect on 28 DIM (*n* = 6/group; *P_TRT_* = 0.003) at 28 DIM, whereby both CONTN and METHS had a reduction in occludin fluorescence intensity (Figure 3B), relative to CONHS (CONTN vs. CONHS: *p* = 0.01; 896.5 vs. 1342.3 ± 119.3; METHS vs. CONHS: *p* = 0.001; 781.3 vs. 1342.3 ± 120.2; Figure 3B).

At 7 DIM (*n* = 8–9/group), K^+^ did not differ (*P_TRT_* = 0.71; Table 1), and while Na^+^ tended to have a treatment effect (*P_TRT_* = 0.07; Table 1), it was not different for the pre-planned pairwise comparisons of interest to this study (*p* ≥ 0.17; Table 1). Yet, there was a treatment effect (*P_TRT_* = 0.01; Table 1) for Na^+^:K^+^ ratio, whereby the ratio tended to be elevated in CONHS, compared to METHS (*p* = 0.07; Table 1). The Na^+^:K^+^ ratio was not different between CONTN and CONHS cows (*p* = 0.14; Table 1). At 28 DIM (*n* = 8–9/group), Na^+^ and the Na^+^:K^+^ ratio were not significantly different between treatments (*P_TRT_* ≥ 0.38; Table 1). There was a treatment effect (*P_TRT_* = 0.05) for the milk concentration of K^+^, whereby it was higher in CONHS, relative to CONTN cows (*p* = 0.04; Table 1). The K^+^ ratio was not different between CONHS and METHS cows (*p* = 0.88; Table 1).

### 3.4. Mammary Gland Cellular Turnover

The mammary gland cellular turnover results are presented in Table 2 (*n* = 6/treatment group). At 8 DIM, nuclei positive for cellular proliferation (Ki67) in the stroma (*P_TRT_* = 0.10) trended toward a treatment effect, whereby it tended to be higher in the METHS cows, relative to the CONHS (*p* = 0.06; Table 2). Stromal cellular proliferation percentages were not different between CONTN and CONHS cows (*p* = 0.78). Yet, the positive nuclei for cellular proliferation in the MEC did not have an overall treatment effect (*P_TRT_* = 0.19); when examining the pre-planned pairwise comparisons, proliferating MEC tended (*p* = 0.09) to be higher in the CONHS, compared to the CONTN cows (Table 2). Lastly, the total proliferating nuclei in the mammary tissue at 8 DIM tended to have a treatment effect (*P_TRT_* = 0.10), although it was not different for the pre-planned pairwise comparisons of interest to this study (*p* ≥ 0.18). At 28 DIM, the proliferation of the mammary tissue was not different between any of our treatments (*P_TRT_* ≥ 0.44). Additionally, there were no differences at both 8 and 28 DIM between our treatments in the nuclei positive for programmed cellular death (TUNEL) in either of the tissue compartments (stroma: *P_TRT_* ≥ 0.23; MEC: *P_TRT_* ≥ 0.32) or in total (*P_TRT_* ≥ 0.48).

## 4. Discussion

The transition period represents one of the most physiologically demanding stages in the life of a dairy cow, characterized by rapid shifts in endocrine status, nutrient partitioning, and tissue remodeling to support the onset of lactation [4,12]. During this window, the mammary gland undergoes extensive structural and functional adaptation while competing with other physiological priorities, including continued fetal growth and systemic metabolic homeostasis [13,39]. Superimposing HS on this already constrained physiological state further challenges the cow’s ability to adequately allocate nutrients and preserve production and tissue integrity. In this study, we used a nutritional approach to investigate whether supplementing RPM to heat-stressed Holstein cows during late gestation and early lactation influences mammary gland development, function, integrity, and remodeling.

From a mammary gland perspective, HS imposes pressure on milk synthesis pathways and glandular remodeling in lactating and non-lactating cows, respectively. Previous studies reported that exposure to HS reduced milk components, downregulated DNA repair and mitochondrial function, and altered mammary gland morphology through smaller alveoli and a greater proportion of connective tissue [40,41,42,43]. Although milk output was not affected by treatments from within the larger study [26], consistent with the literature, the EHB cows displayed mammary microstructural alterations in early lactation, which were characterized by a reduced number of alveoli, increased epithelial cell layer thickness and larger alveolar lumens, and overall larger alveoli, relative to CONTN cows, indicating disrupted structural remodeling during the transition period. It is now recognized that HS-induced reductions in DMI explain approximately 35–50% of the decline in milk yield and components [16,17,44], suggesting direct effects of HS on mammary metabolism and cellular function beyond feed intake impacts. Under HS conditions, the physiological system is forced to reprioritize limited nutrient and metabolic resources, resulting in compromised mammary productivity and integrity through changes in gene expression, cellular turnover, and exfoliation of mammary epithelial cells into milk [45]. Heat stress during late gestation imposes physiological constraints on dry cows that may impair fetal growth and compromise subsequent lactational performance, including reduced milk protein and fat synthesis, altered glucose metabolism, and diminished lactose-driven milk volume [26].

Interestingly, providing RPM during the transition period mitigated several of the HS-induced morphological alterations. Relative to CONHS cows, methionine-supplemented cows in the current study exhibited a greater number of alveoli and smaller mammary epithelial cell layers and alveolus lumens. These findings suggest a partial restoration of normal mammary architecture and indicate that methionine may support the tightly regulated processes of mammary remodeling and secretory differentiation. Collectively, the structural changes observed in the EHB-exposed cows point toward a compromised capacity of the tightly regulated processes governing the mammary gland maturation into a fully high-functioning, high-output secretory phenotype, whereas RPM supplementation appears to partially alleviate these constraints imposed by HS.

This structural vulnerability induced by HS also extends to the mammary epithelial barrier, which functions to separate blood and milk constituents. Barrier integrity is preserved through tight junction complexes connecting alveolar epithelial cells that regulate paracellular permeability and uphold the structural and functional stability of the mammary gland tissue [46]. Studies examining the impacts of HS on mammary epithelial permeability are limited [40]. However, previous research has shown that HS exposure can cause the structural integrity of mammary epithelial cell tight junctions to degrade, resulting in increased permeability and barrier leakage of lactose in Holstein cows [47]. Moreover, in mouse models, HS leads to the damage of tight junction structures and alters the expression of associated tight junction genes and proteins [48]. In the present study, mammary epithelial barrier function appeared to be modestly affected by HS, but responses differed between structural and functional indicators and were most evident early in lactation. The tendency for an elevated Na^+^:K^+^ ratio in CONHS cows, relative to METHS, at 7 DIM is consistent with a transient increase in mammary permeability under HS and may indicate that acute HS has more severe effects on barrier function. Whereas the lower ratio observed in METHS, relative to CONHS, suggests that methionine supplementation may partially support functional epithelial integrity during this critical period by causing a tight junction closure early in lactation. However, by 28 DIM, the absence of treatment differences in Na^+^:K^+^ ratio indicates a recovery or compensation of barrier function as lactation progressed and may simply indicate that under chronic HS, cows prioritize and can maintain barrier integrity.

Despite these functional patterns, these outcomes do not fully align with changes observed at the protein and gene level in the current study. The abundance of occludin, one of the most abundant tight junction proteins, was reduced in methionine-supplemented cows, relative to CONHS, at 8 DIM and at 28 DIM; both CONTN and METHS cows had reduced occludin abundance, both relative to CONHS cows. Additionally, the lack of differences in gene expression of ZO1 and OCLDN and a relatively stable Na^+^:K^+^ ratio point to alterations in tight junction functional dynamics. Taken together, these findings suggest that both HS and methionine supplementation modulate tight junction remodeling rather than inducing overt mammary epithelial barrier failure or disruption. The apparent discrepancy between structural (occludin) and functional (Na^+^:K^+^ ratio) markers highlights the dynamic nature of tight junction regulation during early lactation, where changes in protein abundance and gene expression from HS and methionine supplementation may reflect an adaptive remodeling process to preserve epithelial integrity under compounded stressors.

Within this context, methionine emerges as a key nutritional regulator with roles that extend beyond its function as a precursor for protein synthesis. Previous work has demonstrated that RPM supplementation enhances mammary gland metabolic activity, supports milk protein synthesis, and modulates pathways related to cellular growth, oxidative balance, and nutrient signaling [28,49,50,51]. Importantly, under HS conditions, methionine supplementation has been shown to partially alleviate production losses and improve indicators of metabolic and mammary function, suggesting a role in supporting tissue resilience during periods of stress. Structural adaptations reported in this manuscript were accompanied by improvements in milk protein concentrations, previously reported from the foundational work involving the same group of cows as these cows within [26], suggesting enhanced secretory function of mammary tissue with supplemental methionine. Supporting this interpretation, METHS cows also exhibited greater expression of the SLC3A2 gene, coding for a key component of the heterodimeric amino acid transport system involved in the cellular uptake of large neutral amino acids, including methionine. Increased mammary SLC3A2 gene expression has previously been associated with greater milk protein yield in bovine somatotropin-treated dairy cows, highlighting its potential role in supporting amino acid availability for milk protein synthesis [52].

Additionally, a previous study [53] found RPM supplemented to HS cows improved the number of cows with a high liver functionality index (LFI), decreased IL-6 and IL-1β responses to an in vitro lipopolysaccharide challenge, and increased hepatic expression of methionine-related genes (*MAT1A* and *EIF4EBP1*). Methionine supplementation during HS has also been shown to increase the protein abundance of phosphorylated mTOR (active mTOR) in the mammary tissue of mid-lactation cows [28], which could explain the greater number of alveoli and stromal proliferation seen in the mammary tissue of METHS cows in our study. However, these improvements in mammary physiology occurred despite a significant downregulation of *MTORC1* or downward trends in *ULK1* and *RPS6* (targets of mTOR) gene expression.

Because mTORC1 activity is regulated primarily through phosphorylation of mTOR and its downstream targets, measuring phosphorylation, not transcript abundance, is the most reliable indicator of pathway activation. As our study did not assess phosphorylation status, interpretations of mTOR involvement must be made cautiously. The observation of increased mammary stromal proliferation alongside downregulated *mTORC1* expression suggests that methionine may not solely signal through the mTOR pathway, but rather through alternative pathways, though our current data cannot determine if methionine acted through or independently of the mTOR pathway.

Given this limitation, the possibility that methionine acted through other growth-related pathways, such as MAPK/ERK or STAT5, should be viewed as a speculative direction for future work rather than a mechanism supported by the present results. While mTORC1 primarily regulates protein translation, the MAPK/ERK and STAT5 pathways are more directly associated with cell proliferation and differentiation. Therefore, supplemental methionine may be prioritizing hyperplasia over hypertrophy, which could explain why we see an increased number of alveoli, smaller epithelial cell layers, and a downregulation of mTORC1-related gene expression pathways in METHS cows. Nevertheless, confirming these mechanisms will require future studies that directly quantify pathway activation, including phosphorylation measurements.

The present study builds on our knowledge of HS physiology by providing evidence that HS alters mammary gland microstructure and cellular dynamics during early lactation, consistent with a gland operating under both metabolic and structural strain. Methionine supplementation did not fully prevent these alterations but appeared to modulate the expression of genes and the abundance of proteins in key pathways involved in cellular growth and metabolism, and structural integrity, particularly as lactation progressed. The upregulation of genes associated with one-carbon metabolism and mTOR signaling at 28 DIM (*AHCY*, *GNMT*, *MTORC1*, *AKT1*, *EIF4BP1*), along with partial normalization of structural features, suggests that methionine may enhance the capacity of the mammary gland to adapt and recover under HS conditions, while protecting protein synthesis, without fully rescuing milk volume [26]. Altogether, these findings support the concept that nutritional interventions such as RPM do not simply “rescue” performance under HS, but rather strengthen the biological resilience of the mammary gland during a critical period of transition. In this study, methionine supplementation contributed to maintaining aspects of mammary structure and metabolic signaling, highlighting its potential as a strategy to support mammary function when cows face compounded physiological stressors.

A final consideration is the limitations of this study that should be considered when interpreting the findings within. First, heat stress was induced using electric heat blankets, which provided a controlled and repeatable thermal stimulus but do not fully replicate the complexity of environmental heat load, including humidity, air speed, and solar radiation. As a result, physiological responses to blanket-induced heat stress may differ from those elicited under natural climatic conditions. However, notably, an increasing number of studies have reported hallmark heat-stress responses (increased respiration rates, decreased dry matter intakes, and increased core body temperatures) following electric blanket-induced heat stress, supporting the utility of this model. Second, although transcript abundance and fluorescence-based protein measures offered insight into molecular and structural responses, the study did not include protein-level validation of key signaling pathways, such as phosphorylation assays for mTOR-related targets, limiting mechanistic interpretation. Finally, long-term production outcomes were not assessed, as the cows enrolled in this study were subsequently assigned to other research projects. This prevented continued monitoring beyond 28 days in milk, and therefore the extent to which the structural and molecular changes observed here translate to sustained performance across the full lactation remains unknown.

## 5. Conclusions

In conclusion, even a mild but sustained HS insult during the transition period is sufficient to trigger structural and metabolic constraints that extend beyond modest reductions in feed intake, ultimately compromising mammary gland remodeling and milk composition. Heat stress disrupted mammary cellular dynamics, slightly increased epithelial permeability, and increased tight-junction protein, indicating an overall reduced mammary resilience. Supplementing heat-stressed transition cows with RPM partially alleviated these effects by mitigating epithelial permeability and tight-junction dysfunction, while modulating key pathways involved in mammary gland cellular growth, amino acid transport, and one-carbon metabolism during early lactation. These findings suggest that methionine supports mammary gland adaptability and resilience under HS, highlighting its potential as a nutritional strategy to mitigate the impact of compounded physiological stress during the transition period.

## Figures and Tables

**Table 1:** Milk concentrations of Na^+^ and K^+^ from Holstein multiparous cows assigned to thermoneutral conditions and fed a control diet or exposed to electric heat blanket-induced heat stress and fed either a control or rumen-protected methionine-supplemented diet during the transition period.

Variable	Treatment ^1^			*p*-Value *	
	CONTN	CONHS	METHS	SEM	TRT
7 DIM					
Na^+^, mg/kg	390.6	375.9	355.5	9.77	0.07
K^+^, mg/kg	1558.3	1583.9	1545.9	32.1	0.71
Na^+^:K^+^ Ratio	0.251 ^b^	0.238 ^b^	0.218 ^a^	0.01	0.01
28 DIM					
Na^+^, mg/kg	326.5	333.1	325.8	11.7	0.89
K^+^, mg/kg	1498.4 ^b^	1643.3 ^a^	1653.5 ^a^	48.4	0.05
Na^+^:K^+^ Ratio	0.206	0.203	0.195	0.01	0.38

^1^ Multiparous Holstein cows were assigned to thermoneutral conditions with a control diet (CONTN; *n* = 9), exposed to artificially induced heat stress with a control diet (CONHS; *n* = 8), or exposed to artificially induced heat stress with rumen-protected methionine supplementation (METHS; *n* = 8; Smartamine^®^ M, Adisseo Inc., France, providing 2.6% Met of metabolizable protein compared with 2.2% in control diets). Diets were fed for 10 weeks (CON vs. MET, −6 weeks prepartum to 4 weeks postpartum) and environmental treatments were applied for 8 weeks (TN vs. HS, −4 weeks prepartum to 4 weeks postpartum). Composite milk samples were collected at 7 and 28 DIM. Heat stress was induced using electric heat blankets (Thermotex Therapy Systems Ltd., Calgary, AB, Canada). * Pre-planned pairwise comparisons included CONTN vs. CONHS and CONHS vs. METHS. Different letters indicate significantly different values (*p* ≤ 0.05). Significance was declared at *p* ≤ 0.05 and tendencies at 0.05 < *p* ≤ 0.10.

**Table 2:** Mammary gland cellular turnover assessed by immunohistochemistry at 8 and 28 DIM from Holstein multiparous cows assigned to thermoneutral conditions and fed a control diet or exposed to electric heat blanket-induced heat stress and fed either a control or rumen-protected methionine-supplemented diet during the transition period.

Variable	Treatment ^1^			*p*-Value *	
	CONTN	CONHS	METHS	SEM	TRT
Cell proliferation [Ki-67]					
8 DIM					
Stroma, %	1.56 ^#^	1.46 ^#^	2.21 *	0.27	0.10
MEC, %	0.28 ^#^	0.48 *	0.45 *	0.08	0.19
Total, %	0.72	0.84	1.10	0.13	0.10
28 DIM					
Stroma, %	1.45	1.98	1.56	0.32	0.50
MEC, %	0.41	0.42	0.55	0.09	0.44
Total, %	0.81	0.97	0.94	0.14	0.67
Cell death [TUNEL]					
8 DIM					
Stroma, %	0.42	0.38	0.46	0.05	0.47
MEC, %	0.70	0.92	0.87	0.11	0.32
Total, %	0.58	0.64	0.67	0.05	0.48
28 DIM					
Stroma, %	0.23	0.14	0.23	0.04	0.23
MEC, %	0.31	0.36	0.29	0.04	0.42
Total, %	0.28	0.26	0.26	0.03	0.90

^1^ Multiparous Holstein cows were assigned to thermoneutral conditions with a control diet (CONTN; *n* = 6), exposed to artificially induced heat stress with a control diet (CONHS; *n* = 6), or exposed to artificially induced heat stress with rumen-protected methionine supplementation (METHS; *n* = 6; Smartamine^®^ M, Adisseo Inc., France, providing 2.6% Met of metabolizable protein compared with 2.2% in control diets). Diets were fed for 10 weeks (CON vs. MET, −6 weeks prepartum to 4 weeks postpartum) and environmental treatments were applied for 8 weeks (TN vs. HS, −4 weeks prepartum to 4 weeks postpartum). Heat stress was induced using electric heat blankets (Thermotex Therapy Systems Ltd., Calgary, AB, Canada). Mammary biopsies were collected at 8 and 28 DIM to assess cellular turnover via immunohistochemical staining of nuclear Ki67 (cell proliferation) and TUNEL (TdT dUTP nick-end labeling for apoptosis) in stromal and mammary epithelial (alveolar) compartments. * Pre-planned pairwise comparisons included CONTN vs. CONHS and CONHS vs. METHS. Different symbols indicate a tendency (*p* ≤ 0.10). Significance was declared at *p* ≤ 0.05 and tendencies at 0.05 < *p* ≤ 0.10.

## Data Availability

The original contributions presented in this study are included in the article. Further inquiries can be directed to the corresponding author.

## Abbreviations

The following abbreviations are used in this manuscript:

HS	Heat stress
MG	Mammary gland
RPM	Rumen-protected methionine
MET	Methionine
CON	Control
METHS	Methionine supplemented, heat stress treatment
CONHS	Control diet, heat stress treatment
CONTN	Control diet, thermoneutrality treatment
DIM	Days in milk
d	Day
min	Minute
SAM	S-adenosyl methionine
mTOR	Mechanistic target of rapamycin
EHB	Electric heat blanket
TN	Thermoneutral
*P_TRT_*	The *p*-value for the treatment effect
MEC	Mammary epithelial cells

## Appendix A

**Table A1:** Primer sequences for genes analyzed using real-time q-PCR in bovine mammary glands.

Gene	Forward Primer	Reverse Primer
Housekeeping Transcripts		
*UXT*	TGTGGCCCTTGGATATGGTT	GGTTGTCGCTGAGCTCTGTG
*ACTB*	CACCTTCACCGTTCCAGTTT	ACTTGCGCAGAAAACGAGAT
*KRT8*	GATGAACCGGAACATCAACC	GCCTGACATCCTTAACAGC
Transcripts of Interest		
*ZO1*	CTTTACGAGCTCCAGGCACT	GGGGTCCTTCCTGTACACCT
*OCLDN*	CACCTGCAGCTACTGGACTCT	GAGCAAAAGCCACAATAACCA
*LALBA*	AAAGACTTGAAGGGCTACGGA	AGATGTTGCTTGAGTGAGGGTT
*CSN1S1*	TACCTGTCTTGTGGCTGTTGC	CCTTTTGAATGTGCTTCTGCTC
*CSN1S2*	GCCTGGACTACTGTCTTCCTTTTA	TCCTCTTCATTTGCGTTCCTTAC
*CSN2*	AGTGAGGAACAGCAGCAAACAG	AGCAGAGGCAGAGGAAGGTG
*CSN3*	TTCAACTGCGGTCTATACTCTAAG	TCAAAAAACTAAATCTGGCATAAAAG
*SLC3A2*	GTGTGGACGGGTTTCAGGTC	CCGATCCTCACTGACGCTCT
*SLC7A5*	GGGTGACGTAGCCAATCTGG	ATCCCCCATAGGCAAAGAGG
*MTR*	TGGAGGGTGCGTCTTTTCTG	TCTTCATCCCAGATGGCGTC
*MAT1A*	TTGTGCAGGTGTCCTATGCC	ACGATAACACCAGGCCGAAG
*AHCY*	CATTGGGCACTTTGACGTGG	CAAGTAGCGATCCACCTGGG
*GNMT*	GAGTGACCTGACCAAGGACG	AGCCGGAACTTACTCAAGCC
*DNMT1*	GCGCCTCAGCTAAAATCAAGG	CCACAAACACCGCATACGAC
*DNMT3A*	GGAGTCACTGGAAGCCCAACC	CATAGATCCAGGTGTGGAGCGG
*MTOR*	ATGCTGTCCCTGGTCCTTATG	GGGTCAGAGAGTGGCCTTCAA
*AKT1*	CACGTGCTCTGGACGCTTC	ATGGCGAGGTTCCACTCAAAC
*ULK1*	CAGAACTACCAGCGCATTGA	GTGCAAAGCCCAAAGGACT
*RPS6*	CTGGGTGAAGAATGGAAGGG	CGAACTCTGCCATGGGTCA
*RPS6KB1*	CAAGCTTGCATGCTAATTTGTCC	TTGAGTCCTGATCATGTCGAAGA
*EIF4EBP1*	TTTGAGATGGACATTTAAAGGGC	CTTGCATAAGGCCTGGCTG

*UXT* = Ubiquitously expressed prefoldin like chaperone; *ACTB* = Beta actin; *KRT8* = Keratin 8; *ZO1* = Tight junction protein 1; *OCLDN* = Occludin; *LALBA* = Alpha-Lactalbumin; *CSN1S1* = Alpha casein S1; *CSN1S2* = Alpha casein S2; *CSN2* = Beta casein; *CSN3* = Kappa casein; *SLC3A2* = Solute carrier family 3 member 2, amino acid transporter; *SLC7A5* = Solute carrier family 7 member 5, amino acid transporter; *MTR* = Methionine synthase; *MAT1A* = Methionine adenosyl transferase 1 A; *AHCY* = Adenosyl homocysteinase; *GNMT* = Glycine N-methyltransferase; *DNMT1* = DNA methyltransferase 1; *DNMT3A* = DNA methyltransferase 3 alpha; *MTOR* = Mechanistic target of rapamycin; *AKT1* = AKT serine/threonine kinase 1; *ULK1* = Unc-51-like autophagy activating kinase 1; *RPS6* = Ribosomal protein 6; *RPS6KB1* = Ribosomal protein S6 kinase B1; *EIF4EBP1* = Eukaryotic translation initiation factor 4E-binding protein 1.

## References

[1] Bernabucci U., Lacetera N., Baumgard L.H., Rhoads R.P., Ronchi B., Nardone A. (2010). Metabolic and hormonal acclimation to heat stress in domesticated ruminants. Animal.

[2] Gorniak T., Meyer U., Sudekum K.H., Danicke S. (2014). Impact of mild heat stress on dry matter intake, milk yield and milk composition in mid-lactation Holstein dairy cows in a temperate climate. Arch. Anim. Nutr..

[3] do Amaral B.C., Connor E.E., Tao S., Hayen M.J., Bubolz J.W., Dahl G.E. (2009). Heat-stress abatement during the dry period: Does cooling improve transition into lactation?. J. Dairy Sci..

[4] Tao S., Bubolz J.W., do Amaral B.C., Thompson I.M., Hayen M.J., Johnson S.E., Dahl G.E. (2011). Effect of heat stress during the dry period on mammary gland development. J. Dairy Sci..

[5] Fabris T.F., Laporta J., Skibiel A.L., Corra F.N., Senn B.D., Wohlgemuth S.E., Dahl G.E. (2019). Effect of heat stress during early, late, and entire dry period on dairy cattle. J. Dairy Sci..

[6] West J.W. (2003). Effects of heat-stress on production in dairy cattle. J. Dairy Sci..

[7] Roth Z. (2020). Influence of heat stress on reproduction in dairy cows—Physiological and practical aspects. J. Anim. Sci..

[8] Gao S.T., Guo J., Quan S.Y., Nan X.M., Fernandez M.V.S., Baumgard L.H., Bu D.P. (2017). The effects of heat stress on protein metabolism in lactating Holstein cows. J. Dairy Sci..

[9] Chanda T., Debnath G.K., Khan K.I., Rahman M.M., Chanda G.C. (2017). Impact of heat stress on milk yield and composition in early lactation of Holstein Friesian crossbred cattle. Bangladesh J. Anim. Sci..

[10] Intergovernmental Panel on Climate Change (IPCC) (2007). Climate Change 2007: Synthesis Report.

[11] Beck H.E., Zimmermann N.E., McVicar T.R., Vergopolan N., Berg A., Wood E.F. (2018). Present and future Köppen-Geiger climate classification maps at 1-km resolution. Sci. Data.

[12] Grummer R.R. (1995). Impact of changes in organic nutrient metabolism on feeding the transition dairy cow. J. Anim. Sci..

[13] Capuco A.V., Akers R.M. (1999). Mammary involution in dairy animals. J. Mammary Gland Biol. Neoplasia.

[14] Drackley J.K. (1999). Biology of dairy cows during the transition period: The final frontier?. J. Dairy Sci..

[15] Overton T.R., Waldron M.R. (2004). Nutritional management of transition dairy cows: 231 Strategies to optimize metabolic health. J. Dairy Sci..

[16] Baumgard L.H., Rhoads R.P. (2012). Ruminant Nutrition Symposium: Ruminant production and metabolic responses to heat stress. J. Anim. Sci..

[17] Xiao Y., Kronenfeld J.M., Renquist B.J. (2020). Feed intake-dependent and -independent effects of heat stress on lactation and mammary gland development. J. Dairy Sci..

[18] Cowley F.C., Barber D.G., Houlihan A.V., Poppi D.P. (2015). Immediate and residual effects of heat stress and restricted intake on milk protein and casein composition and energy metabolism. J. Dairy Sci..

[19] Broderick G.A., Kowalczyk T., Satter L.D. (1970). Milk production response to supplementation with encapsulated methionine per OS or casein per abomasum. J. Dairy Sci..

[20] Schwab C.G., Broderick G.A. (2017). A 100-year review: Protein and amino acid nutrition in dairy cows. J. Dairy Sci..

[21] Lee C., Hristov A.N., Cassidy T.W., Heyler K.S., Lapierre H., Varga G.A., de Veth M.D., Patton R.A., Parys C. (2012). Rumen-protected lysine, methionine, and histidine increase milk protein yield in dairy cows fed a metabolizable protein-deficient diet. J. Dairy Sci..

[22] Osorio J.S., Ji P., Drackley J.K., Luchini D., Loor J.J. (2013). Supplemental Smartamine M or MetaSmart during the transition period benefits postpartal cow performance and blood neutrophil function. J. Dairy Sci..

[23] Zhou Z., Vailati-Riboni M., Trevisi E., Drackley J.K., Luchini D.N., Loor J.J. (2016). Better postpartal performance in dairy cows supplemented with rumen-protected methionine compared with choline during the peripartal period. J. Dairy Sci..

[24] Batistel F., Arroyo J.M., Bellingeri A., Wang L., Saremi B., Parys C., Trevisi E., Cardoso F.C., Loor J.J. (2017). Ethyl-cellulose rumen protected methionine enhances performance during the periparturient period and early lactation in Holstein dairy cows. J. Dairy Sci..

[25] Elsaadawy S.A., Wu Z., Wang H., Hanigan M.D., Bu D. (2022). Supplementing ruminally protected lysine, methionine, or combination improved milk production in transition dairy cows. Front. Vet. Sci..

[26] Davidson B.D., Zambon A.A., Guadagnin A.R., Hoppmann A., Larsen G.A., Sherlock D.N., Luchini D., Arriola Apelo S.I., Laporta J. (2024). Rumen-protected methionine supplementation during the transition period under artificially induced heat stress: Effects on cow-calf performance. J. Dairy Sci..

[27] Lynch C.J., Adams S.H. (2014). Branched-chain amino acids in metabolic signaling and insulin resistance. Nat. Rev. Endocrinol..

[28] Coleman D.N., Vailati-Riboni M., Pate R.T., Aboragah A., Luchini D., Cardoso F.C., Loor J.J. (2022). Increased supply of methionine during a heat-stress challenge in lactating Holstein cows alters mammary tissue mTOR signaling and its response to lipopolysaccharides. J. Anim. Sci..

[29] Dai W.T., White R.R., Liu J.X., Liu H.Y. (2018). Seryl-tRNA synthetase-mediated essential amino acids regulate β-casein synthesis via cell proliferation and mammalian target of rapamycin (mTOR) signaling pathway in bovine mammary epithelial cells. J. Dairy Sci..

[30] Xing Z., Tu B.P. (2025). Mechanisms and rationales of SAM homeostasis. Trends Biochem. Sci..

[31] Field S.L., Davidson B.D., Hoerl A.F., Dado-Senn B., Hernandez L.L., Laporta J. (2023). Amplifying local serotonin signaling prior to dry-off hastens mammary gland involution and redevelopment in dairy cows. J. Dairy Sci..

[32] Schneider C.A., Rasband W.S., Eliceiri K.W. (2012). NIH image to ImageJ: 25 years of image analysis. Nat. Methods.

[33] Skibiel A.L., Dado-Senn B., Fabris T.F., Dahl G.E., Laporta J. (2018). In-utero exposure to thermal stress has long-term effects on mammary gland microstructure and function in dairy cattle. PLoS ONE.

[34] Field S.L., Marrero M.G., Dado-Senn B., Skibiel A.L., Ramos P.M., Scheffler T.L., Laporta J. (2021). Peripheral serotonin regulates glucose and insulin metabolism in Holstein dairy calves. Domest. Anim. Endocrinol..

[35] Clarida (2026). Reference Gene Finder (Version 2026.07.0). https://app.clarida.bio/reference-gene-finder/genorm.

[36] Vandesompele J., De Preter K., Pattyn F., Poppe B., Van Roy N., De Paepe A., Speleman F. (2002). Accurate normalization of real-time quantitative RT-PCR data by geometric averaging of multiple internal control genes. Genome Biol..

[37] Sorensen A., Muir D.D., Knight C.H. (2001). Thrice-daily milking throughout lactation maintains epithelial integrity and thereby improves milk protein quality. J. Dairy Res..

[38] Herve L., Quesnel H., Greuter A., Hugonin L., Merlot E., Le Floc’H N. (2023). Effect of the supplementation with a combination of plant extracts on sow and piglet performance and physiology during lactation and around weaning. J. Anim. Sci..

[39] Bauman D.E., Currie W.B. (1980). Partitioning of nutrients during pregnancy and lactation: A review of mechanisms involving homeostasis and homeorhesis. J. Dairy Sci..

[40] Tao S., Orellana R.M., Weng X., Marins T.N., Dahl G.E., Bernard J.K. (2018). Symposium Review: The influences of heat stress on bovine mammary gland function. J. Dairy Sci..

[41] Dado-Senn B., Skibiel A.L., Fabris T.F., Dahl G.E., Laporta J. (2019). Dry period heat stress induces microstructural changes in the lactating mammary gland. PLoS ONE.

[42] Perez-Hernandez G., Ellett M.D., Banda L.J., Dougherty D., Parsons C.L.M., Lengi A.J., Daniels K.M., Corl B.A. (2024). Cyclical heat stress during lactation influences the microstructure of the bovine mammary gland. J. Dairy Sci..

[43] Perez-Hernandez G., Ellett M.D., Pokhrel B., Parsons C.L.M., Corl B.A., Daniels K.M. (2025). Transcriptomic changes induced by controlled cyclical heat stress in the bovine mammary gland during lactation. J. Dairy Sci. Commun..

[44] Wheelock J.B., Rhoads R.P., VanBaale M.J., Sanders S.R., Baumgard L.H. (2010). Effects of heat stress on energetic metabolism in lactating Holstein cows. J. Dairy Sci..

[45] Leduc A., Souchet S., Gelé M., Le Provost F., Boutinaud M. (2021). Effect of feed restriction on dairy cow milk production: A review. J. Anim. Sci..

[46] Nguyen D.A.D., Neville M.C. (1998). Tight junction regulation in the mammary gland. J. Mammary Gland Biol. Neoplasia.

[47] Weng X., Monteiro A.P.A., Guo J., Li C., Orellana R.M., Marins T.N., Bernard J.K., Tomlinson D.J., DeFrain J.M., Wohlgemuth S.E. (2018). Effects of heat stress and dietary zinc source on performance and mammary epithelial integrity of lactating dairy cows. J. Dairy Sci..

[48] Zheng Y., Zhao Y., He W., Wang Y., Cao Z., Yang H., Wang W., Li S. (2022). Novel organic selenium source hydroxy-selenomethionine counteracts the blood-milk barrier disruption and inflammatory response of mice under heat stress. Front. Immunol..

[49] Pate R.T., Luchini D., Murphy M.R., Cardoso F.C. (2020). Effects of rumen-protected methionine on lactation performance and physiological variables during a heat stress challenge in lactating Holstein cows. J. Dairy Sci..

[50] Junior V.C., Lopes F., Schwab C.G., Toledo M.Z., Collao-Saenz E.A. (2021). Effects of rumen-protected methionine supplementation on the performance of high production dairy cows in the tropics. PLoS ONE.

[51] Costa T.C., Nascimento K.B., Danés M.A.C., Gionbelli M.P., Duarte M.S. (2025). Rumen-protected methionine for dairy and beef cattle: Current perspectives on methionine role, supplementation strategies, metabolism, health, and performance. Front. Anim. Sci..

[52] Sciascia M., Pacheco D., McCoard S.A. (2015). Administration of exogenous growth hormone is associated with changes in plasma and intracellular mammary amino acid profiles and abundance of the mammary gland amino acid transporter SLC3A2 in mid-lactation dairy cows. PLoS ONE.

[53] Guadagnin A.R., Tabor E., Davidson B.D., Guenther M.C., Larsen G., Sherlock D.N., Luchini D., Arriola Apelo S.I., Laporta J. (2026). Peripartum heat stress and rumen-protected methionine supplementation: Effects on immune, metabolic, and inflammatory status of Holstein cows and her offspring. J. Dairy Sci..

